# Student Perceptions of Competition in Medical Education: Comparing Individual and Collaborative Approaches

**DOI:** 10.1111/tct.70423

**Published:** 2026-04-22

**Authors:** Edward Finch

**Affiliations:** ^1^ Division of Clinical Medicine, School of Medicine and Population Health The University of Sheffield Sheffield UK

**Keywords:** collaboration, competition, graduate outcomes, medical education, student perceptions, teaching methods, welfare

## Abstract

**Background:**

Medical education is often perceived as a highly competitive environment, with competition influencing both student motivation and graduate outcomes. Previous research has reported mixed findings on the impact of competitive teaching methods, particularly on academic performance and broader graduate outcomes, such as student well‐being and teamwork. A greater understanding of how students perceive competition, including the role of collaborative competition, is needed to inform teaching practices that support both learning and professional development. This study aimed to explore how medical students perceive competition in the learning environment, focusing on the contrast between individual and collaborative competition and their perceived effects on professional development.

**Methods:**

In 2020, a questionnaire incorporating both closed‐ended and open‐ended questions was distributed to all 1384 registered medical students at the University of Sheffield. Quantitative data were collected via Likert scales and analysed statistically, while qualitative free‐text responses underwent thematic analysis. A convergent parallel design was used to integrate quantitative and qualitative findings, allowing a comprehensive understanding of student perspectives.

**Results:**

Eighty‐five students responded, with 38 providing free‐text comments. Students reported similar levels of competitiveness across sex and year of study, and perceived themselves as less competitive than their peers. Collaborative competition was preferred over individual competition and was perceived to support key graduate outcomes by maintaining the benefits of individual competition while mitigating its negative aspects.

**Conclusion:**

These findings reinforce the perceived competitive nature of medical education and highlight the importance of considering student perceptions in shaping teaching practices.

## 1 Background

Competition is an inherent part of everyday life, shaping experiences in education, the workplace and beyond. Often so embedded that it goes unnoticed [1]. Medical education is no exception [2, 3]. While competition can serve as a powerful academic motivator, encouraging students to strive for excellence, its impact is not universally positive [4, 5, 6, 7, 8]. In particular, individual competition tends to reward academic knowledge acquisition, sometimes at the expense of broader competencies such as communication, empathy and teamwork [4, 5].

This tension is particularly relevant in the context of modern medical education, which is shifting from a sole focus on academic knowledge towards the development of holistic graduate outcomes [9, 10]. From a patient's perspective, communication is a key determinant they consider when evaluating how satisfied they are with their doctor [11]. On top of this, deficiencies in teamwork and interpersonal skills are often linked to adverse events, including medical errors and patient mortality [12]. As such, the modern doctor is not only a skilled clinician but also a collaborative team member who demonstrates empathy towards both colleagues and patients [13, 14].

The increasingly competitive postgraduate job market further adds pressure to medical students, who already face high rates of stress, burnout and substance misuse [15, 16]. These challenges highlight the need for thoughtful curriculum design. Student perceptions, reflected in tools such as the National Student Survey, are central to shaping medical education [15]. To prepare graduates for a profession that values both individual excellence and teamwork, educators must understand how students experience competition and collaboration. These insights can guide strategies that support the development of both resilient competitors and empathetic team players.

This apparent contradiction underscores an important area of scholarly interest: how competition, particularly within medical education, can be structured to enhance, rather than hinder, the attainment of holistic graduate outcomes. Traditional individual competition fosters self‐reliance and personal resilience [1], but may also increase anxiety and reduce willingness to engage in shared learning [7, 8]. In contrast, collaborative learning environments, where success depends on mutual effort, have been associated with lower stress levels and improved psychological well‐being [1, 6]. Collaborative competition, in which teams, rather than individuals compete, has been proposed as a model that combines benefits of both approaches [17, 18]. However, empirical research directly comparing medical students' perceptions of the effects of individual versus collaborative competition on graduate outcomes, particularly within the United Kingdom (UK) context, remains limited.

Medical educators are therefore tasked with equipping future doctors not only with clinical expertise but also with the interpersonal and emotional skills necessary to thrive in a demanding profession [3, 13, 16]. Exploring student perceptions of competition can provide crucial insights into designing educational strategies that can better support this dual preparedness.

This study therefore sought to investigate how medical students perceive competition in the learning environment, with particular attention to the contrast between how individual and collaborative forms and their relation to professional development.

*This study therefore sought to investigate how medical students perceive competition in the learning environment, with particular attention to the contrast between how individual and collaborative forms and their relation to professional development.*

This study aimed to address the following research questions:

1. How do medical students perceive competition in the learning environment?
2. How do medical students perceive the impact of individual competition on graduate outcomes?
3. How do medical students' perceptions of collaborative competition compare to individual competition in relation to graduate outcomes?

## 2 Methods

### 2.1 Study Design

This study employed a convergent parallel design, in which quantitative and qualitative data were collected simultaneously, analysed independently and subsequently merged to inform interpretation. The findings were then presented together to address each research question in turn, with the narrative of the results emerging through the integration of qualitative analysis, which guided the interpretation of the quantitative data [19].

This design was grounded in a pragmatic paradigm, which supports the complementary use of quantitative and qualitative approaches to explore the relationships that exist between the many layers of the multifaceted research questions [19].

Quantitative data were collected using Likert scales and analysed using statistical methods. Participants were also invited to provide additional free‐text comments regarding the use of competition within the learning environment. This allowed participants to elaborate on their responses and explore aspects not captured by the quantitative data. The full questionnaire used in this study is provided in Supporting Information.

The questionnaire was developed from key themes identified in the literature review, mapped to the UK General Medical Council's and the University of Sheffield's graduate outcomes and reviewed by faculty with expertise in medical education to support content validity [9, 10]. These outcomes included motivation to learn, ability to work under pressure, critical thinking, feedback on learning, enjoyment of learning, stress and anxiety levels, coping mechanisms, communication skills and teamwork. The questionnaire was then piloted with students to ensure face validity. All 1384 registered medical students at the University of Sheffield were invited to participate. Data collection occurred over a two‐week period in March 2020. The questionnaire was distributed via a post on Minerva (the official virtual learning platform) and relevant Facebook groups, with reminders sent 1 week and 1 day before the deadline.

### 2.2 Data Analysis

Quantitative data were analysed using the Statistical Package for the Social Sciences, version 26. Mann–Whitney *U* tests were performed for subgroup analyses to assess for statistically significant differences between the medians of two independent groups. The Kruskal–Wallis *H* test was performed to test for statistically significant differences between medians of more than two independent groups. A Wilcoxon signed rank test was used to assess the statistical difference between paired samples; it was therefore used to compare two sets of Likert scores that came from the same participants. Significance was determined by *p* values at the 5% level (*p* < 0.05). For clarity in presenting general patterns within the data, related Likert‐scale categories were combined when reporting overall trends. Specifically, *strongly agree* and *agree* were combined, as were *disagree* and *strongly disagree*. Similarly, response options indicating that an item ‘contributed in a somewhat positive way’ and ‘contributed in a positive way’ were combined, as were those indicating a ‘somewhat negative’ or ‘negative’ contribution.

Qualitative data were analysed thematically by the author using an inductive approach, following the six‐step framework described by Braun and Clarke [20]. We acknowledge that the author's lived experience may influence data interpretation. A reflexive approach, including regular reflective review meetings with a senior faculty member experienced in medical education research, was employed to enhance rigour, ensure trustworthiness and authentically represent participants' perspectives.

### 2.3 Ethical Approval

This project was approved by the University of Sheffield, Department of Biomedical Sciences Ethics Review Committee (Reference Number: 029547). All participants provided written informed consent for the use of their questionnaire responses in this research.

## 3 Results

The teaching environment questionnaire received responses from 85 (6.1%) of the 1384 registered medical students, with participants representative of all years in the five‐year program at the University of Sheffield Medical School at the time of the study (see Table 1). The responses, when compared with admissions data, demonstrated that the results were representative of the current medical student cohort, with no significant differences in male‐to‐female ratio (*p* = 0.14) or graduate‐entry to undergraduate‐entry split (*p* = 0.52).

**TABLE 1:** Participants' demographics. *N* = 85.

Demographics		Number	Percentage
Sex^a^	Male	28	33%
	Female	57	67%
Undergraduate‐entry vs. graduate‐entry^a^	Undergraduate‐entry	50	82%
	Graduate‐entry	37	12%
Stage of medical school training	Phase 1 (Year 1)	18	21%
	Phase 2 (Year 2/3)	18	21%
	Phase 3a (Year 3/4)	17	20%
	Phase 3b (Year 4)	10	12%
	Phase 4 (Year 5)	12	14%
	Intercalating^b^	10	12%

^a^ Compared with admissions data from the previous 3 years, the study population showed no statistically significant differences in demographic characteristics.

^b^ Intercalating refers to medical students who are currently taking a year out of the standard medical curriculum to pursue an additional qualification.

Of the 85 participating students, 38 (44.7%) provided free text responses. Through our analysis, we identified eight themes, each mapped to the research questions outlined in Table 2, together with a description and illustrative quotations capturing the essence of the theme.

**TABLE 2:** Qualitative themes and illustrative quotations of free text comments. *N* = 38.

Theme	Description	Illustrative quotations
**Research Question 1: How do medical students perceive competition in the learning environment?**		
Motivational benefits	Competition can enhance motivation, focus and consistent effort, helping students track progress and push themselves academically.	‘I think personally it gives me an underlying level of good stress and motivation to work hard to earn my place and not become complacent, which I see as a good thing.’ (S6) ‘Competition and ranking is good for improving standards of learning and provoking ambition amongst students.’ (S76)
Psychological costs	Competition can generate stress and pressure, negatively affecting wellbeing and potentially harming peer relationships.	‘Competition can motivate me to learn more or work harder however I think it can have very negative impacts on mental health and could also harm friendships/relationships with peers.’ (S18) ‘I believe that competition increases an individual's desire to learn more and be better. However, I do understand that an overly competitive curriculum may be detrimental to those who may not do as well.’ (S10)
Drivers of competition	Students' competitive drive stems from intrinsic factors and structural elements of the learning environment, such as ranking systems.	‘I know peers that are competitive with the grading system, and others that are not bothered by it. I think a certain level of competition with yourself is positive in order to better yourself.’ (S32) ‘A lot of my motivation comes from not wanting to be ranked last or in the bottom 50’ (S28)
Assessment influence	Competition is constructive when low‐stakes, anonymous or self‐directed, but can be harmful when high‐stakes or ranking‐focused.	‘I feel like competition in the medical school learning environment should be encouraged but should not be used as a means of assessing students.’ (S11) ‘I believe competition can be good and bad depending on how it is done. When it comes to competition regarding quizzes … as long as it is anonymous in a lecture theatre, I believe they are a great way for the students to test themselves for particular topics and have an immediate comparison to their peers. However, competition is detrimental when it is fixed on a particular number (e.g., end of year ranking for exams).’ (S24)
**Research Question 2: How do medical students perceive the impact of individual competition on graduate outcomes?**		
Comparative feedback	Students view individual competition as a source of comparative feedback that highlights areas for improvement, but it can also produce negative effects, making direct comparison potentially harmful.	‘I think competition is motivating and helps me stay focussed. It helps me compare my progress with others and lets me know what I need to work on more.’ (S7) ‘I think competition within learning environments at the medical school can be a great source of anxiety and stress for many students. For example, scoring lower than your peers on a quiz/assessment can cause a student to worry about their academic progress and doubt themselves, rather than motivate them to study more.’ (S81)
Misalignment with professional values	Individual competition may encourage self‐focused behaviours, which students recognise as conflicting with the teamwork and collaboration essential for clinical practice.	‘Competing as an individual, can induce more stress in a person and I have seen people become slightly more hostile towards their peers, so they can “show off” how much better they are.’ (S16) ‘It can be detrimental when students are driven to “win” in order to feed their egos rather than focussing on the real goal ‐ positive patient outcomes ‐ which necessarily comes as a result of team work.’ (S17) ‘When working as a doctor it's much more important to work as a team ‐ it does not matter if your colleague is better than you, you all need to work together for the good of the patients.’ (S28)
**Research Question 3: How do medical students' perceptions of collaborative competition compare to individual competition in relation to graduate outcomes?**		
Facilitate student interaction	Collaborative competition promotes knowledge sharing and helps students develop the interpersonal skills essential for clinical practice.	‘The competition should be used to bring out the best in students and then assessments should be used when students have worked competitively with others and then together to ensure all students feel prepared and have worked together to collate knowledge.’ (S11) ‘A team competitive activity in the first semester would be useful as it would facilitate interaction in the first term and if it was towards the end of first semester, could be a way of consolidating knowledge.’ (S75)
Wider skills developed	Collaborative competition develops leadership, organisation and teamwork abilities, helping students navigate challenges in clinical practice.	‘In a team environment, I believe it could have a beneficial effect in terms of organisation, leadership and a more positive learning environment where everyone can contribute with their strengths and boost their weak attributes.’ (S16) ‘Team competition can be good, but is sometimes frustrating when group members have different expectations and put in different effort. However this gives experience for life and work.’ (S32)

*Note:* Each comment was assigned a code, with ‘S’ denoting Student, followed by a number corresponding to the order in which the response was received.

### 3.1 Research Question 1

How do medical students perceive competition in the learning environment?

These findings indicate that students perceive themselves to be equally competitive regardless of sex (*U* = 724.5, *p* = 0.43) or year of study (*χ* ^2^ = 2.587, *p* = 0.76). Students reported being competitive in both an academic (61, 71.8%) and social (56, 65.9%) environment, as well as with themselves (78, 91.8%). Although the 70 students (82.4%) agreed they were a competitive person, 33 students (38.9%) disagreed with the statement ‘I am a competitive person compared to my peer group’. This suggests that, despite most students perceiving themselves as competitive, they view their peer group as more competitive.

Further insight into the quantitative results can be given by the four qualitative themes relating to research question one. The **motivational benefits** and **psychological costs** of competition in the learning environment, in terms of student welfare and peer interaction, are reflected in students' qualitative responses.

Competition can motivate me to learn more or work harder however I think it can have very negative impacts on mental health and could also harm friendships/relationships with peers. (S18)

Students described the **drivers for competition** they experienced. While many students acknowledged being competitive, in the comments they also perceived their peers to be more competitive than themselves, suggesting that competition is often viewed as a relative experience. It emerges as a significant feature of the learning environment, shaped by both peer‐related and self‐directed pressures.

I know peers that are competitive with the grading system, and others that are not bothered by it. I think a certain level of competition with yourself is positive in order to better yourself. (S32)

These dynamics appear to stem not only from students' intrinsic motivation but also from structural aspects of the educational environment, particularly during assessments and especially when ranked results are published.

A lot of my motivation comes from not wanting to be ranked last or in the bottom 50 ‐ I know that has no affect or importance but that's my aim and that helps me to revise. (S28)

The way **assessment influences** the application of competition within the curriculum also shapes how it is perceived. Students view competition as constructive when it is formative, low‐stakes, anonymous or self‐directed, but potentially harmful when it is summative, high‐stakes or ranking‐focused.

I believe competition can be good and bad depending on how it is done. When it comes to competition regarding quizzes … as long as it is anonymous in a lecture theatre, I believe they are a great way for the students to test themselves for particular topics and have an immediate comparison to their peers. However, competition is detrimental when it is fixed on a particular number (e.g., end of year ranking for exams). (S24)

### 3.2 Research Question 2

How do medical students perceive the impact of individual competition on graduate outcomes?

**FIGURE 1:**
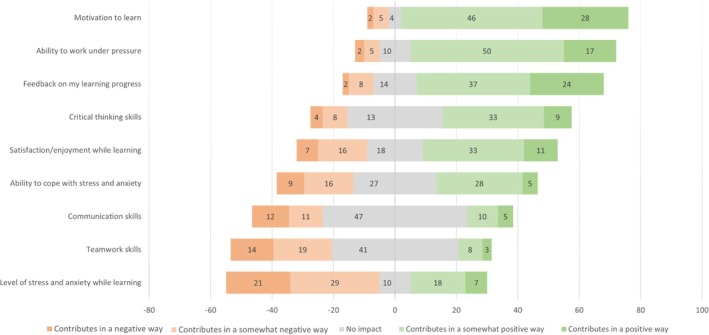
A Likert plot showing medical student responses (count) to how individual competition impacts different graduate outcomes (ordered most positive to most negative). *N* = 85.

Students were asked to score the impact of individual competition on the graduate outcomes using a 5‐point Likert scale from *contributes in a negative way* to *contributes in a positive way* (Figure 1). Aspects of learning in which students thought competition contributed in an overall positive way were motivation to learn, 74 (87.0%); ability to work under pressure, 67 (78.8%); feedback on their learning progress, 61 (71.8%); satisfaction/enjoyment while learning, 44 (51.8%); critical thinking skills', 42 (49.4%); and ability to cope with stress and anxiety, 33 (38.8%). Fifty students (58.8%) felt that an individual competitive environment contributed in a negative way to their stress and anxiety while learning. Of the 85 participants, 47 (55.3%) felt individual competition had no impact on developing communication skills and 41 (48.2%) felt it had no impact on developing teamwork skills. Despite the positive element competition offers, 46 students (54.1%) felt that individual competition should not be encouraged in medical education.

In the qualitative responses students reported that individual competition provides **comparative feedback**, helping them identify areas for improvement, but it can also lead to negative effects, making feedback through direct comparison potentially harmful and influencing their readiness for professional practice after graduation.

I think competition within learning environments at the medical school can be a great source of anxiety and stress for many students. For example, scoring lower than your peers on a quiz/assessment can cause a student to worry about their academic progress and doubt themselves, rather than motivate them to study more. (S81)

Individual competition may encourage self‐focused behaviours, which students recognise as **misaligned with professional values**, such as teamwork and communication skills essential for clinical practice. Students placed greater value on these graduate outcomes than on academic motivation or ability.

It can be detrimental when students are driven to ‘win’ in order to feed their egos rather than focussing on the real goal ‐ positive patient outcomes ‐ which necessarily comes as a result of teamwork. (S17)

### 3.3 Research Question 3

How do medical students' perceptions of collaborative competition compare to individual competition in relation to graduate outcomes?

**FIGURE 2:**
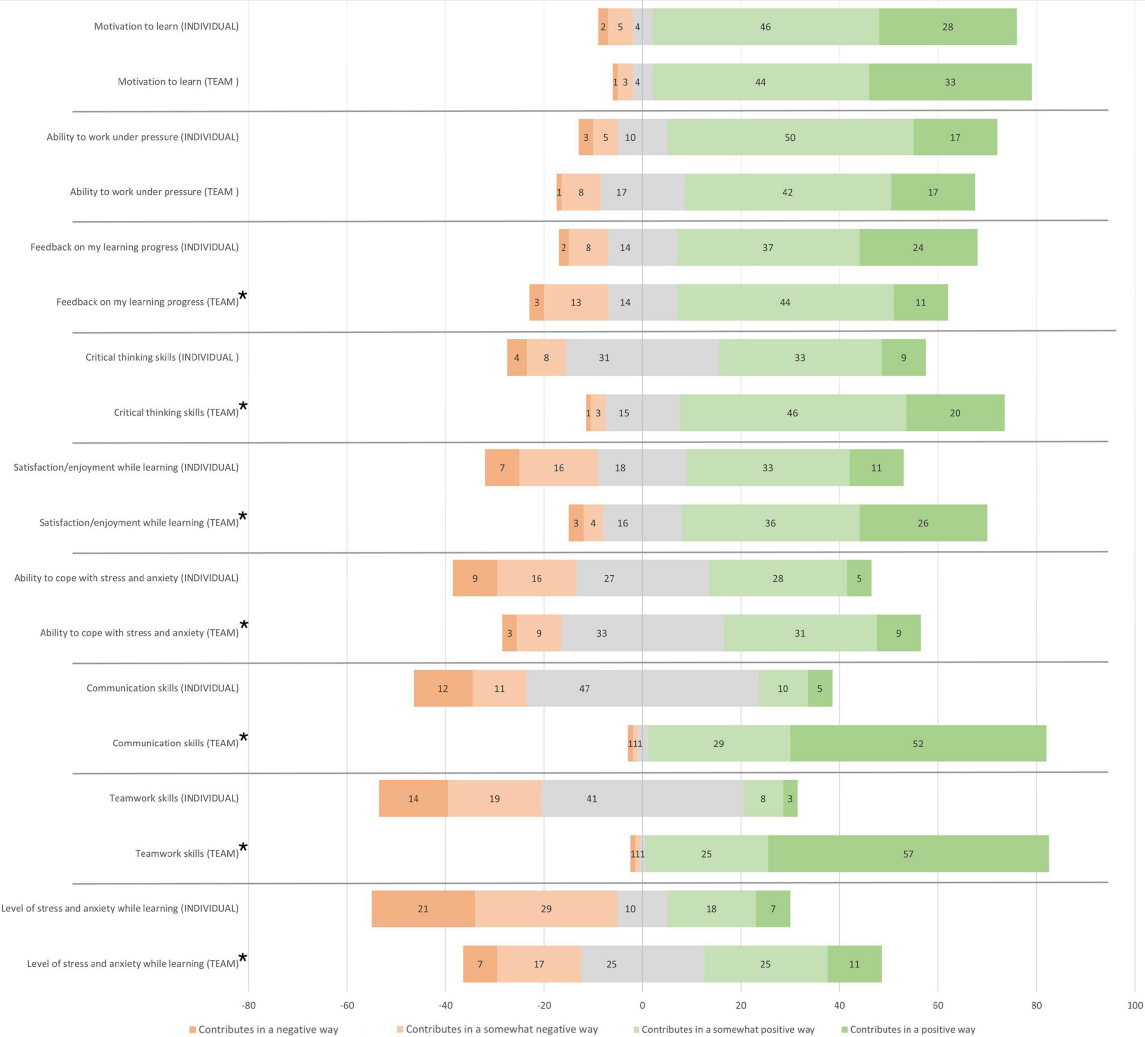
A Likert plot showing medical student responses (count) to how collaborative competition as a team impacts graduate outcomes compared with individual competition. *N* = 85. Statistically significant results are denoted with an asterisk (*p* < 0.05).

To answer this question, students were then asked to give their opinion on the same graduate outcomes as above but from the perspective of competing collaboratively as part of a team, as opposed to competing as an individual (Table 3). Percentages of overall positive or negative impact were used to evaluate the difference (Figure 2).

**TABLE 3:** Comparison of medical student responses to how collaborative competition as a team impacts graduate outcomes compared with individual competition. *N* = 85.

Graduate outcomes	Individual median (IQR)	Team median (IQR)	Wilcoxon standardised test statistic, *Z*	*p*	Effect size
Motivation to learn	4 (4–5)	4 (4–5)	1.443	0.149	0.16
Critical thinking skills	3 (3–4)	4 (4–4)	4.601	< 0.01*	0.50
Ability to work under pressure	4 (4–4)	4 (3–4)	−0.637	0.524	0.07
Communication skills	3 (2–3)	5 (4–5)	7.689	< 0.01*	0.83
Teamwork skills	3 (1–3)	5 (4–5)	7.712	< 0.01*	0.84
Level of stress and anxiety while learning	2 (2–4)	3 (2–4)	3.613	< 0.01*	0.39
Ability to cope with stress and anxiety	3 (2–4)	3 (3–4)	2.566	< 0.01*	0.28
Feedback on my learning progress	4 (3–5)	4 (3–4)	−2.078	< 0.038*	0.23
Satisfaction/enjoyment while learning	4 (2–4)	4 (3–5)	3.776	< 0.01*	0.41

*Note:* The responses to each statement were scored using a Likert scale ranging from 1 to 5 (1 = *Not confident at all*, 5 = *Very confident*). Statistically significant results are denoted with an asterisk (*p* < 0.05).

Abbreviation: IQR = interquartile range.

The results of this study show that students felt that collaborative competition maintains the benefits of individual competition, with no significant difference between the voting for motivation to learn (*Z* = 1.443, *p* = 0.149) and ability to work under pressure (*Z* = −0.637, *p* = 0.524), with both continuing to contribute in a positive way, with 77 (90.6%) and 59 (69.4%) students, respectively. The overall positive contribution when competing individually for critical thinking, students' ability to cope with stress and anxiety and satisfaction/enjoyment while learning was further enhanced by competing as a team: critical thinking increased from 42 (49.4%) to 66 (77.6%) (*Z* = 4.601, *p* < 0.01); ability to cope with stress and anxiety increased from 33 (38.8%) to 40 (47.1%) (*Z* = 2.566, *p* < 0.01); and satisfaction/enjoyment while learning increased from 44 (51.8%) to 62 (72.9%) (*Z* = 3.776, p < 0.01).

Forty‐seven students (55.3%) went from thinking individual competition had no impact to 81 students (95.1%) reporting an overall positive effect on communication when competing collaboratively (*Z* = 7.689, *p* < 0.01). A similar effect was also seen with teamwork; 41 students (48.2%) thinking it had no impact when competing individually, to having an overall positive effect, 81 students (96.5%) when competing collaboratively (*Z* = 7.712, p < 0.01). Overall, both individual and collaborative competition contributed in a positive way towards receiving feedback while learning. However, more students, 61 (71.8%), felt competing as an individual had a positive impact on their feedback rather than competing as a team, 55 (64.7%) (*Z* = −2.078, *p* = 0.038).

When students were asked about the type of competition that they would like to see more of, 46 students (54.1%) disagreed with the statement ‘I would like to see more individual competition in the medical educational environment’ compared with 46 students (54.1%) who agreed with the statement ‘I would like to see more team competition in the medical educational environment’.

Students reported that collaborative competition fosters greater **student interaction** and encourages knowledge sharing rather than hoarding. By not relying on direct individual comparison, it is perceived as less personal, which may explain how team‐based competition can retain the benefits of competition while reducing its drawbacks.

The competition should be used to bring out the best in students and then assessments should be used when students have worked competitively with others and then together to ensure all students feel prepared and have worked together to collate knowledge. (S11)

Lastly, collaborative competition is perceived to foster **wider skills development** than individual competition, including outcomes not initially considered in the questionnaire, such as organisational and leadership skills, which help students navigate challenges in clinical practice.

In a team environment, I believe it could have a beneficial effect in terms of organisation, leadership and a more positive learning environment where everyone can contribute with their strengths and boost their weak attributes. (S16)

Responses to the additional survey items are presented in Tables S1 and S2.

## 4 Discussion

This study aimed to explore medical students' perceptions of competition within their learning environment and assess the impact of individual versus collaborative competition on graduate outcomes. Based on the available evidence, this research is the first to use a convergent parallel approach that quantitatively evaluates competition in medical education and follows this with a qualitative exploration of its perceived impact. This design enhances the practical relevance of the findings, providing a more comprehensive understanding of competition's role in medical education.

The findings demonstrate that students prefer collaborative competition because it fosters teamwork, knowledge sharing and mutual support, rather than promoting self‐focused behaviours or pressure to outperform peers. This highlights the potential of collaborative competition to retain the motivating aspects of competition while mitigating its negative effects, making it a more sustainable model for medical education.

*The findings demonstrate that students prefer collaborative competition because it fosters teamwork, knowledge sharing and mutual support, rather than promoting self‐focused behaviours or pressure to outperform peers.*

The results support previous literature indicating that medical education is a competitive learning environment [2, 3]. The findings also align with studies suggesting that individual competition has a mixed impact on students [4, 5, 6, 7, 8] and collaborative learning fosters positive graduate outcomes, such as teamwork and communication skills [17, 18].

In addition to academic and team‐oriented outcomes, the impact of competition on students' well‐being is crucial. Negative experiences during medical school, such as stress and burnout, can have long‐lasting consequences on doctors' mental health and career satisfaction [2, 16]. Previous research has shown that early‐career stress significantly influences long‐term well‐being, job satisfaction and even patient care [21, 22]. Student perspectives suggest that the teaching methods used in medical education can influence how students engage with competition and develop graduate outcomes. This research highlights the importance of applying competition thoughtfully within the curriculum and underscores the need for early interventions that foster an environment in which students can thrive both academically and personally.

*Student perspectives suggest that the teaching methods used in medical education can influence how students engage with competition and develop graduate outcomes.*

The data were collected in March 2020, during the COVID‐19 pandemic, when traditional collaborative learning was disrupted, resulting in reduced opportunities for peer comparison and potentially influencing students' perceptions of competition [23]. Participants noted that peer comparison in a collaborative setting was important for receiving feedback, suggesting that post‐pandemic hybrid learning could benefit from incorporating collaborative competition.

## 5 Limitations

This study has several limitations. The sample was relatively small, drawn from a single UK institution, and participation was voluntary, which may have introduced non‐response bias. Although anonymity reduced confidentiality concerns and encouraged authentic responses, the emotive nature of competition may have attracted students with stronger views, potentially influencing the findings [19]. These factors limit the generalisability of the results to the wider student population and to other institutions or countries. Finally, qualitative coding was conducted by a single researcher, which may have introduced bias. The steps taken to mitigate this are detailed in the methodology.

Future research is needed to determine whether these perceived benefits are realised in practice and how collaborative competition can be effectively integrated to enhance learning outcomes and student development. Further work should also explore the long‐term impact on graduate outcomes, including career success and job satisfaction. In addition, perceptions of competition may vary across cultures and contexts, [1, 16, 21] and studies could investigate whether the findings of this research are consistent in different cultures and educational contexts.

## 6 Conclusion

In conclusion, both the quantitative and qualitative findings highlight the importance of understanding medical students' perceptions of competition and its potential impact on graduate outcomes. Student perspectives suggest that integrating collaborative competition into medical education could provide an academically motivating yet supportive learning environment, fostering teamwork and reducing stress and promoting professional growth. Further research should examine if these benefits occur in applied settings, their lasting impact on graduate outcomes, and whether similar findings are evident in varied educational settings.

## Author Contributions

**Edward Finch:** conceptualization, investigation, writing – original draft, methodology, validation, visualization, writing – review and editing, formal analysis, data curation, project administration.

## Funding

The author has nothing to report.

## Ethics Statement

This project was ethically approved by the Department of Biomedical Sciences ethics review procedure (Reference Number 029547).

## Conflicts of Interest

The author declares no conflicts of interest.

## Supporting information




**Data S1:** Supporting Information.

**Table S1:** Student perception on the impact of competition in the learning environment.
**Table S2:** Student perceptions of the relative importance of doctor attributes.

## Data Availability

The data that support the findings of this study are available from the corresponding author upon reasonable request.
