# Supplementary figures and images for: Student Perceptions of Competition in Medical Education: Comparing Individual and Collaborative Approaches
Source: Clin Teach. 2026 Apr 22;23(3):e70423. doi: 10.1111/tct.70423 (PMC13101406; doi:10.1111/tct.70423)

**Supplementary Material - Teaching Environment Questionnaire**


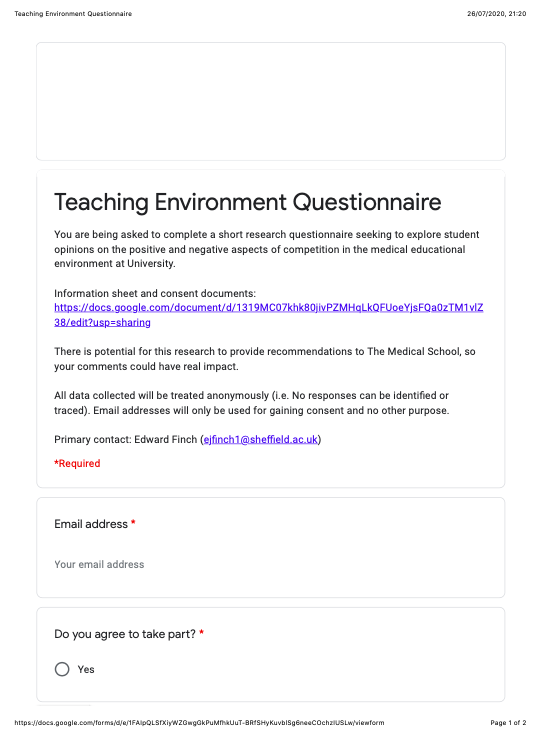

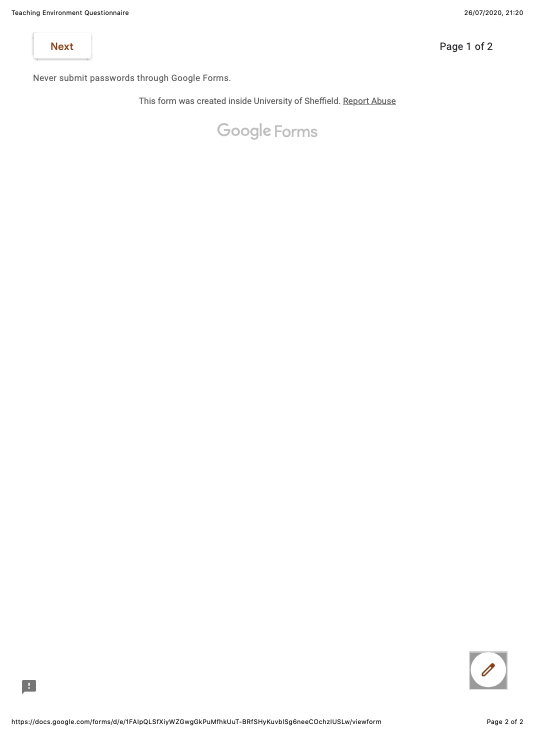


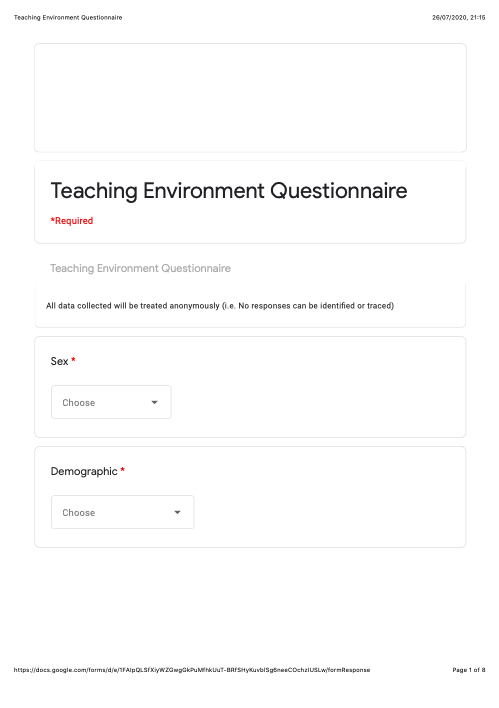


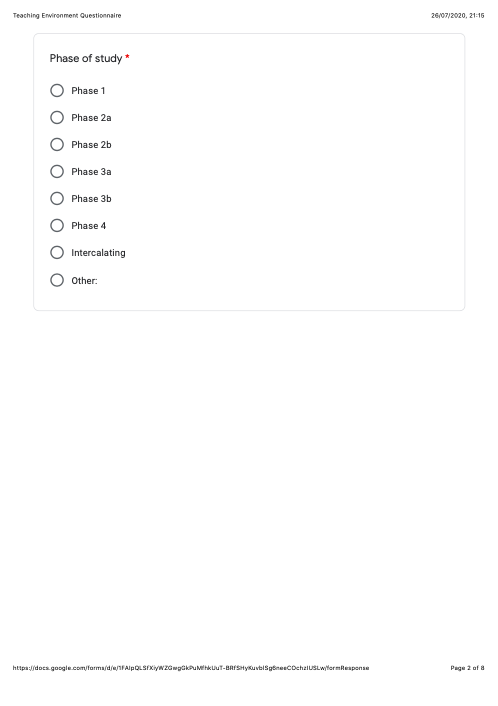


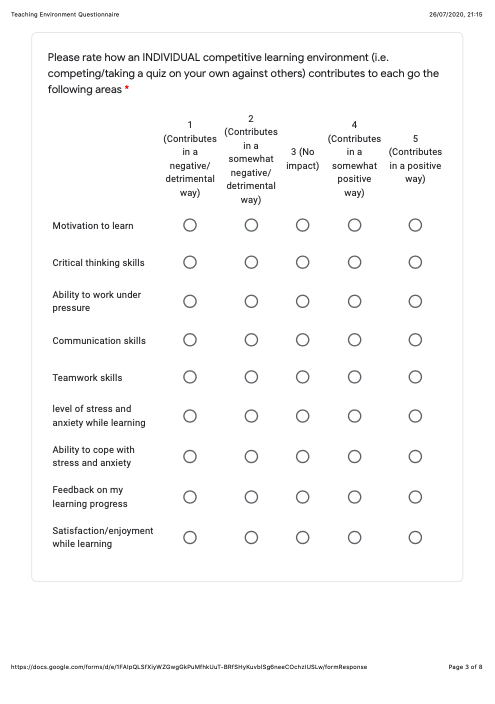


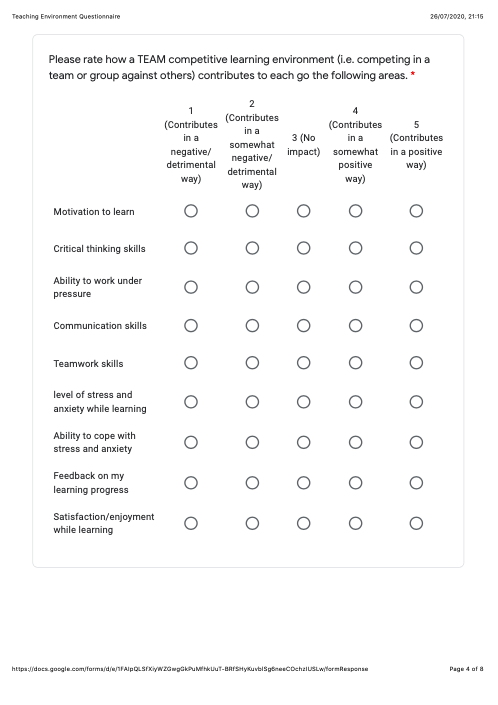


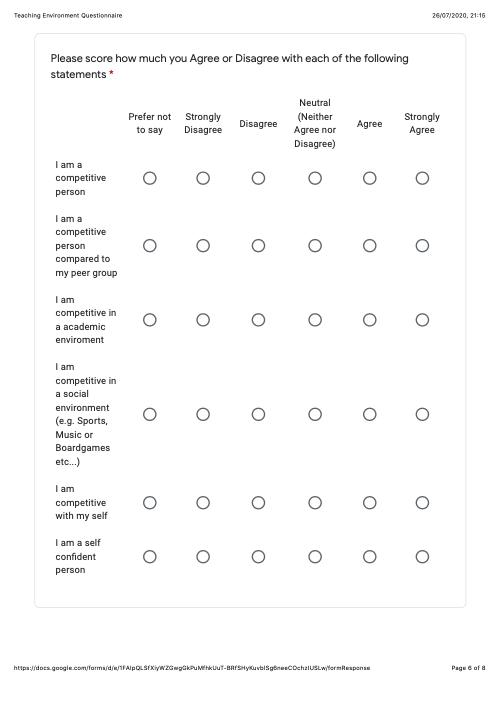


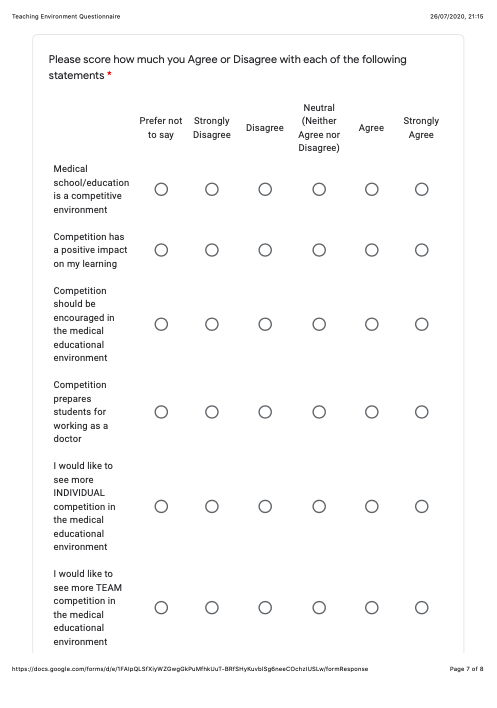


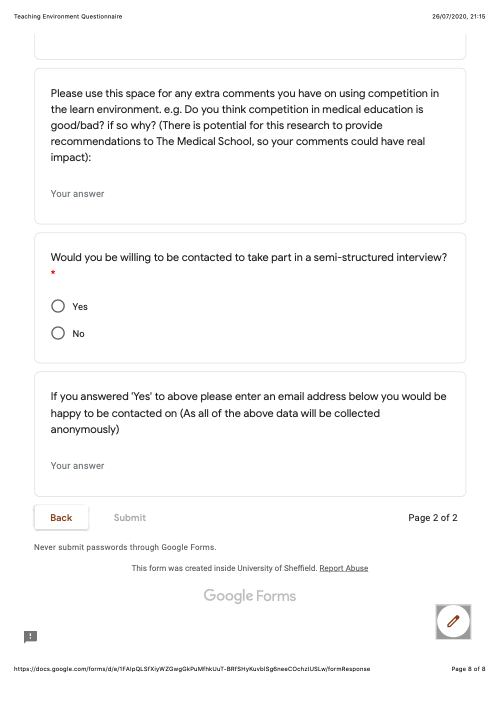


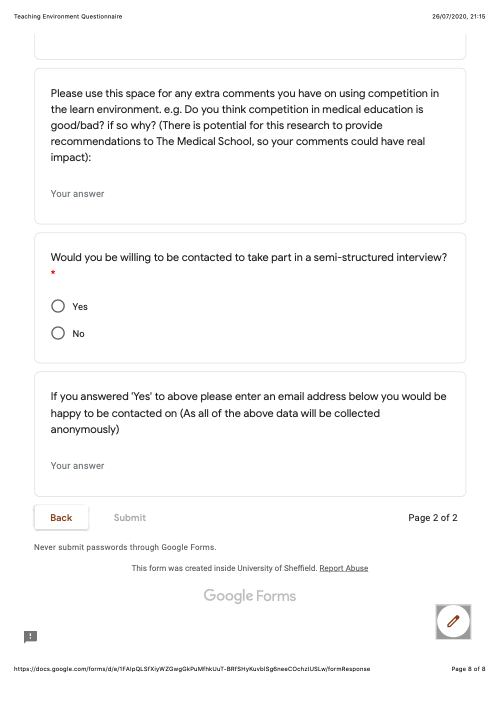

Supplement: Supplementary file 1 — Data S1: Supporting Information. [file TCT-23-e70423-s002.docx]
