# Supplementary material for: Student Perceptions of Competition in Medical Education: Comparing Individual and Collaborative Approaches
Source: Clin Teach. 2026 Apr 22;23(3):e70423. doi: 10.1111/tct.70423 (PMC13101406; doi:10.1111/tct.70423)
Supplement: Supplementary file 2 — Table S1: Student perception on the impact of competition in the learning environment. Table S2: Student perceptions of the relative importance of doctor attributes. [file TCT-23-e70423-s001.docx]

### Supplementary table 1: Student perception on the impact of competition in the learning environment.

| **Statement** | **Median** | **Interquartile range** |
| --- | --- | --- |
| I am a competitive person | 4 | 4-4 |
| I am a competitive person compared to my peer group | 3 | 2-4 |
| I am competitive in an academic environment | 4 | 3-4 |
| I am competitive in a social environment | 4 | 2-5 |
| I am competitive with myself | 5 | 4-5 |
| I am a self-confident person | 3 | 2-4 |
| Medical school is a competitive environment | 4 | 4-5 |
| Competition has a positive impact on my learning | 3 | 2-4 |
| Competition should be encouraged in the medical educational environment | 2 | 2-3 |
| Competition prepares students for working as a doctor | 2 | 2-4 |
| I would like to see more INDIVIDUAL competition in the medical educational environment | 2 | 2-3 |
| I would like to see more TEAM competition in the medical educational environment | 4 | 3-4 |

**Note:** The responses to each statement were scored using a Likert scale ranging from 1 to 5 (1= Strongly disagree, 5 = Strongly Agree).

### Supplementary table 2: Student perceptions of the relative importance of doctor attributes.

| **Core Attributes of a doctor** | **Mean rank** |
| --- | --- |
| Core medical knowledge | 2.49 |
| Teamwork | 3.08 |
| Problem solving | 2.39 |
| Ability to work under pressure | 2.04 |

**Note:** Students ranked the four attributes of a doctor on a scale from 1 (least important) to 4 (most important).
